# Reliability of Capillary Complete Blood Count in Children With Acute Gastroenteritis

**DOI:** 10.3389/fped.2021.715576

**Published:** 2021-08-10

**Authors:** Hanna Wielińska-Wiśniewska, Jan K. Nowak, Michał Da̧browski, Paula Szydłowska, Mariusz Szczepanik, Katarzyna Cichocka, Patrycja Krzyżanowska-Jankowska, Jarosław Walkowiak

**Affiliations:** Department of Pediatric Gastroenterology and Metabolic Diseases, Poznan University of Medical Sciences, Poznan, Poland

**Keywords:** capillary, complete blood count, full blood count, leukocytosis, accuracy, acute diarrhea in children

## Abstract

**Background:** To assess the reliability of complete blood count (CBC) in the capillary blood of children with acute gastroenteritis (AGE), with a focus on leukocytes.

**Methods:** This was a retrospective cross-sectional study. Complete blood count was compared between the capillary and venous blood in children admitted to a pediatric gastroenterology department with primary diagnosis of AGE (ICD-10 A09, A08.0, A08.2). Capillary blood was obtained in the emergency room and venous blood was sampled in the ward shortly thereafter during peripheral intravenous line placement.

**Results:** One hundred and forty children were included. The mean (±SD) age and weight of patients were 3.0 ± 2.9 years and 16 ± 9 kg; 26% had leukocytosis. The mean difference between obtaining results of capillary and venous blood tests was 2 ± 1 h. Area under the receiver operating characteristic curve (AUC) for the identification of leukocytosis using the capillary blood was 0.98 (95% CI 0.96–1.0). The sensitivity and specificity were 86 and 98%, respectively (accuracy 95%). The positive and negative predictive values were 94 and 95%, respectively. The intraclass correlation coefficient revealed high concordance between capillary and venous CBC measurements (leukocyte count 0.94, hemoglobin 0.88, erythrocyte count 0.77, hematocrit 0.79, platelet count 0.90). Matched pairs comparisons revealed marginally higher erythrocytes (difference of medians: 0.2 T/L), hemoglobin (0.3 g/dL), hematocrit (1.0%), and platelets (9 G/L) in the capillary blood.

**Conclusion:** Capillary CBC is useful in detecting leukocytosis in children with AGE.

## Introduction

Acute gastroenteritis (AGE) is defined by ESPGHAN as a decrease in the consistency of stools and/or an increase in their frequency, with or without fever or vomiting. AGE remains a leading cause of pediatric hospitalizations, especially in children below 3 years of age (1).

Apart from clinical signs and symptoms of dehydration, physicians in emergency departments often rely on results of blood tests when making decisions on hospital admission. The tests may include electrolyte levels, blood gas (pH, bicarbonates, base excess) and also a complete blood count (CBC), which helps to rule out more severe conditions. In the ambulatory setting, the collection of capillary blood is also less invasive for the patient than venous blood sampling. However, dehydration, small fingers and the poor capillary network may cause technical difficulties in capillary blood sampling among pediatric patients and reduce diagnostic accuracy (2).

These limitations make the value of capillary CBC uncertain, especially in the emergency room in the course of differential diagnosis of vomiting, diarrhea, and/or fever in children. Therefore, the aim of this study was to assess the reliability of capillary CBC in children with AGE.

## Methods

This cross-sectional study was conducted in Karol Jonscher Clinical Hospital of Poznan University of Medical Sciences, Poznan, Poland. The data were retrospectively collected from electronic health records covering the years 2006–2015. The inclusion criteria comprised: (a) a primary diagnosis of AGE (codes according to ICD-10: A09, A08.0, and A08.2), (b) hematological and electrolyte measurements in the capillary blood in the emergency room and in the venous blood, which were obtained during venipuncture on admission to the hospital, and (c) the time between receiving the results of the two measurements shorter than 4 h. The same methodology was used in our previous study of capillary blood potassium levels (3). The study focused on AGE in order to provide a well-defined group of patients with high availability of the required data.

The capillary and the venous blood samples were analyzed using Sysmex XS-800i and Sysmex XN-1000, respectively (Sysmex Europe GmbH, Norderstedt, Germany). The cut-off for leukocytosis was age-dependent: 20 G/L (7 days−12 months), 13 G/L (1–6 years), 12 G/L (7–12 years), and 10 G/L (>12 years). The lower limit of the reference range for hemoglobin concentrations was: 13.5 g/dL (1st month), 10.0 g/dL (2nd month), 9.5 g/dL (3–5 months), 10.0 g/dL (6–8 months), 10.5 g/dL (9th month), 11.0 g/dL (10–23 months), 10.9 g/dL (2–6 years), 12.0 g/dL (7–12 years), 12.0 g/dL (>12 years female), 14.0 g/dL (>12 years male). Data for leukocyte types or RBC characteristics (e.g., mean corpuscular volume) were not available.

We used Statistica 12 software (Statsoft Inc., Tulsa, USA) to perform the statistical tests, calculate the areas under the curve (AUC) of the receiver operating characteristic, and draw Bland-Altman plots (4). Receiver operating characteristic curve calculations used binary outcome data obtained by comparing venous blood leukocyte count against the above-mentioned age-specific thresholds. The predictor variable was obtained by dividing capillary blood leukocyte concentration by upper limit of the norm for the age. The Youden's index was used to determine the optimal cut-off. The same was done for hemoglobin. The Kolmogorov-Smirnov test was used to test for normality. Forward stepwise regression was used to investigate the influence of age and base excess (as a proxy for dehydration) and other factors on the absolute error of capillary blood measurements.

The intraclass correlation coefficient was calculated in an Excel 2016 spreadsheet (Microsoft, Redmond, USA). The design of the project was inspired by STAndards for Reporting Diagnostic accuracy studies (STARD) (5). The study was approved by the Bioethical Committee at Poznan University of Medical Sciences (541/15). It adhered to the tenets of the revised Declaration of Helsinki.

## Results

Out of 1,080 children with the primary diagnosis of acute gastroenteritis, 140 met the inclusion criteria (90 with A09, 40 with A08.0; age range 0–16 years). The group characteristics, including the mean ± SD values of basic hematological parameters, are presented in Table 1.

**Table 1:** Group characteristics.

**Parameter**	
Sample size	140
Age, years	3.0 ± 2.9
Body weight on admission, kg	16 ± 9
Sex	54.3% male (*n* = 76) 45.7% female (*n* = 64)
Time from admission to capillary results, min	11 ± 42
Time from admission to venous results, min	137 ± 58
Time from capillary to venous results, min	126 ± 48
**Capillary/venous**
Hemoglobin, g/dL	12.8 ± 1.4/12.4 ± 1.2
Anemia	5% / 5% (*n* = 7)
RBC count, T/L	4.7 ± 0.5/4.5 ± 0.4
Hematocrit, %	37 ± 4/35 ± 3
WBC count, G/L	11.1 ± 5.9/11.2 ± 5.7
Leukocytosis	24% (*n* = 33)/26% (*n* = 36)
Platelet count, G/L	312 ± 99/322 ± 111
Comorbidities	39%
Respiratory tract, % of cases with comorbidities	60%
**Symptom frequency**
Vomiting	81%
Diarrhea	64%
Fever	35%
Lethargy	26%
Symptom duration before admission, days	2.0 ± 1.3
Rotaviral infection	49% (52 out of 106)
Capillary base excess of extracellular fluid	−6.9 ± 4.0

*Means ± SD are presented*.

**Figure 1:**
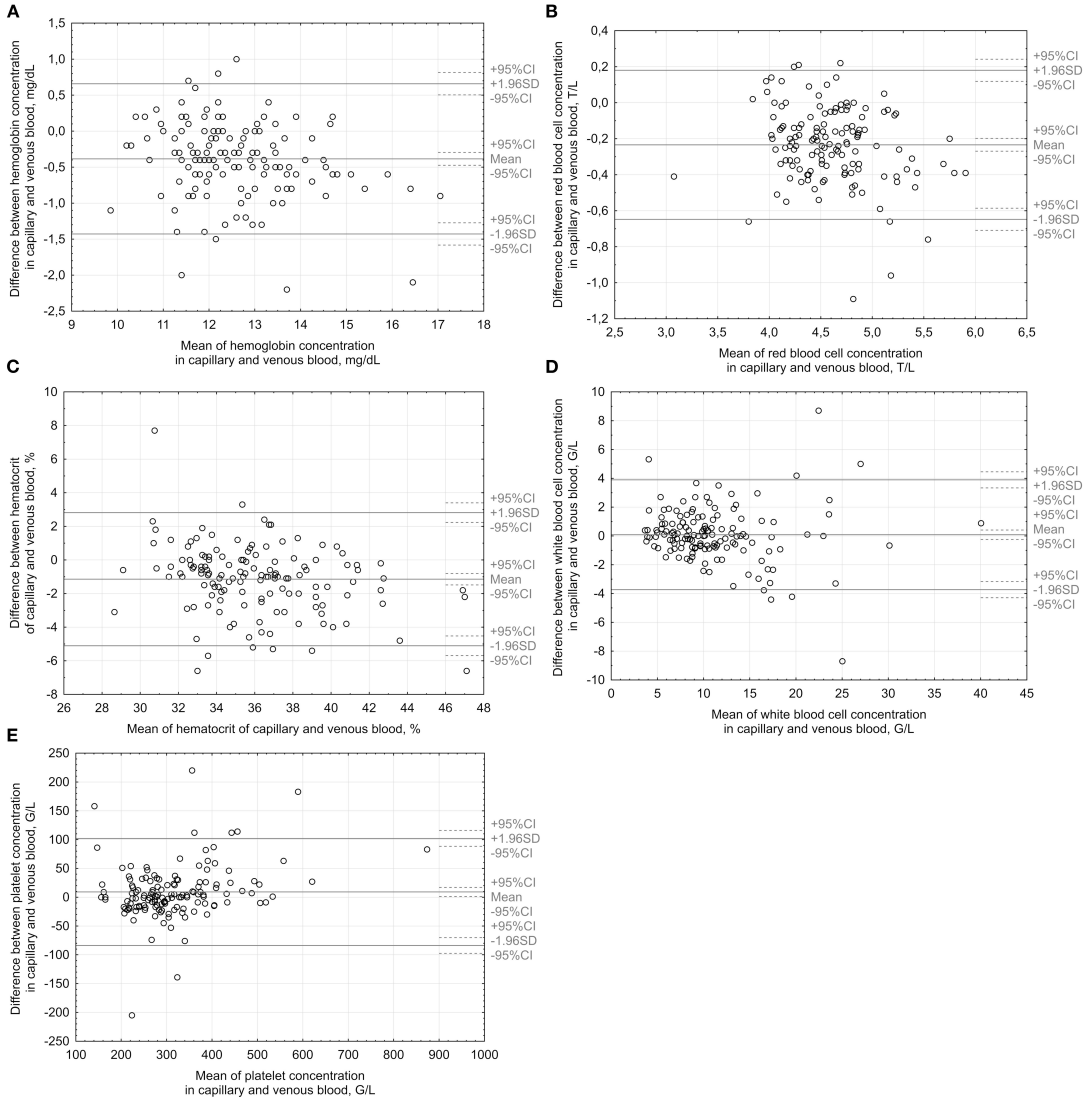
Bland-Altman plots illustrate the agreement between capillary and venous measurements of hemoglobin **(A)**, red blood cells **(B)**, hematocrit **(C)**, leukocytes **(D)** and platelets **(E)**. 1 G/L = 1 000/μL; 1 T/L = 1 000 000/L.

Matched pairs analysis revealed a small yet consistent bias of capillary hemoglobin, RBC count, and hematocrit toward higher values (Table 2). The capillary measurements' absolute error was low (Table 2). The agreement of measurements in the capillary and the venous blood is illustrated in Bland-Altman plots (Figure 1; Table 3).

**Table 2:** Matched-pairs comparisons of hematologic parameters in capillary and venous blood.

**Parameter**	**Capillary blood**	**Venous blood**	**P**	**Absolute error**	**Absolute error 5th−95th percentile**
Hemoglobin, g/dL	12.5 (11.9–13.5)	12.2 (11.5–13.1)	<10^−12^	0.4 (0.4–0.6)	0–1.3
RBC count, T/L	4.7 (4.4–4.9)	4.5 (4.2–4.7)	<10^−19^	0.2 (0.1–0.4)	0–0.5
Hematocrit, %	35.9 (33.9–38.8)	34.9 (33.1–37.5)	<10^−9^	1.2% (0.6–2.4)	0.1–5.2
WBC count, G/L	9.8 (7.2–13.4)	10.0 (7.4–13.5)	0.66	0.9 (0.4–1.8)	0–4.0
Platelet count, G/L	300 (247–360)	291 (246–379)	0.04	18 (8—33)	1–112

*Median values (1st−3rd quartiles) are presented. The Wilcoxon test was applied*.

**Table 3:** Correlation coefficients for hematological parameters measured in capillary and venous blood; *p* < 0.01 for all correlations.

**Parameter**	**Pearson's r**	**Spearman's ρ**	**ICC**
Hemoglobin	0.92	0.89	0.88
RBC count	0.90	0.89	0.77
Hematocrit	0.85	0.82	0.79
WBC count	0.94	0.94	0.94
Platelet count	0.91	0.89	0.90

*ICC, intraclass correlation coefficient*.

AUC for identification of leukocytosis using the measurements in the capillary blood was 0.98 (95% CI 0.96–1.0). The sensitivity and specificity were 86 and 98%, respectively (accuracy 95%). The positive and negative predictive values were 94 and 95%, respectively.

AUC for detecting hemoglobin levels corresponding to anemia—not for diagnosing anemia since the patients were dehydrated—was 0.86 (95% CI 0.68–1.00). The sensitivity was 71% and the specificity 98%, accuracy 97%, positive predictive value 71%, negative predictive value 98%. In seven children, venous hemoglobin levels corresponding to the diagnosis of anemia were found. Five of these were also identified by the capillary blood analysis. However, two false-positive read-outs were also provided by capillary blood analyzers.

Venous hematocrit did not correlate with an absolute error of any of the hematological measurements. Of the five analyzed hematological variables, only RBC count weakly correlated with the difference in time between receiving the results of assessments performed in the venous and the capillary blood (Spearman's ρ = 0.19, *p* = 0.02). However, forward stepwise regression did not confirm any important, independent relationships between the relative or absolute error of capillary CBC measurements and the aforementioned time difference, age, sex, pH, base excess of extracellular fluid, or urea. A few weak associations (*R*^2^ < 0.20) were found using regression: the relative error of capillary hematocrit associated with body weight, hemoglobin correlated with body weight and age, and absolute capillary white blood cell (WBC) count error related to height.

Measurements were grouped time-wise to identify potential systematic bias related to hypothetical changes in sampling or equipment operation practice: 2007–2011 (*n* = 40), 2012–2013 (*n* = 59), 2014–2015 (*n* = 41). The Kruskal-Wallis non-parametric analysis of variance was used to compare the distributions of hematologic parameters between the three groups. Two significant differences were found: WBC concentrations were lower in the years 2007–2011 than from 2012 onwards (venous *p* = 0.004 and capillary *p* = 0.006, respectively). Because the effect was consistent in the venous and in the capillary blood, we did not ascribe it to a measurement error and assumed that it does not disturb the measures of reliability. Comparison of the two periods (2007–2011 vs. 2012–2015) did not identify differences in age, body weight, the length of stay or other major factors that might be associated with WBC count. However, a shift toward afternoon and evening admissions was noted in the later years (1 a.m. ± 4 h vs. 4 p.m. ± 5 h previously, *p* = 0.005). The percentage of admissions after 6 p.m. increased from 13 to 36%.

## Discussion

This is the first study to assess the reliability of capillary CBC in the setting of AGE. Capillary blood, as well as venous blood measurement, were performed in a specialized medical laboratory, using automated hematology analyzers. This pragmatic study had a sizeable convenience sample, employed a statistical approach suitable for determining diagnostic value, and attempted compensation for potential confounders (2). Overall, the differences between the hemoglobin concentration, hematocrit, WBC, and RBC count in the venous and the capillary blood were practically insignificant.

A recently published study comprehensively reviewed the value of capillary vs. venous blood in the assessment of anemia and called for further research (2). Although the results were inconclusive, most of the identified studies revealed a higher hemoglobin concentration in the capillary blood compared with the venous blood. Our results confirm a marginally higher hemoglobin in capillary vs. venous samples. Capillary CBC has a lower sensitivity in the diagnosis of anemia and should not be used to this aim, at least unless its limitations are thoughtfully considered (6–9).

Multiple biological factors influencing the accurateness of capillary blood hemoglobin concentration measurement in AGE were found. This study revealed that body weight and age slightly affected accuracy of capillary hemoglobin and hematocrit measurements. Previous research pointed toward the dependence of the value of capillary CBC on sex, which could be mediated by the vasoconstrictive effect of androgens. As in males capillaries contain less RBCs, hence less hemoglobin (10). This study found no effects of sex on obtained differences between capillary and venous CBC in AGE.

The method of capillary blood sample collection also significantly affects results. Hemoglobin concentration in pooled sample drops is higher than in the single drop of capillary blood (6). Therefore, the pooled sample method (also used herein) more precisely reflects the actual hemoglobin. This study indicates that capillary CBC may be more precise in the estimation of hemoglobin concentration among younger children and those with lower body weight, despite increased technical difficulties in sampling capillary blood. Similar results were obtained previously (11).

Platelets (PLT) and WBC count belong to essential CBC measurements. PLT levels are known to be significantly underestimated in the capillary samples (12–16). On the contrary, WBC count is higher in the capillary than in reference venous blood (12, 14, 17–20). This analysis did not reveal analogous differences.

The main aim of this retrospective work was to determine the reliability of capillary CBC in AGE, which results in dehydration. No relationships were found between base excess corrected for the extracellular fluid (BE-ECF) and the accuracy of capillary CBC. BE-ECF can only estimate the level of dehydration, which is challenging to assess correctly even using complex clinical questionnaires.

In summary, despite being prone to biological, technical and environmental confounders, capillary CBC is useful in detecting leukocytosis in children with AGE (21).

## Data Availability Statement

The raw data supporting the conclusions of this article will be made available by the authors, without undue reservation.

## Ethics Statement

The study was approved by the Bioethical Committee at Poznan University of Medical Sciences (541/15). Written informed consent from the participants' legal guardian/next of kin was not required to participate in this study in accordance with the national legislation and the institutional requirements.

## Author Contributions

HW-W, JN, MD, PS, PK-J, and JW: conceptualization. HW-W, JN, MD, and PS: data curation and formal analysis. HW-W, JN, MS, KC, PK-J, and JW: investigation. JN: project administration. HW-W and JN: writing—original draft. MD, PS, MS, KC, PK-J, and JW: writing—review and editing. JW: funding acquisition and supervision. All authors have approved the final article.

## Conflict of Interest

JN reports personal fees from Norsa Pharma, a grant from Biocodex Microbiota Foundation, and non-financial support from Nutricia outside the submitted work. JW reports personal fees and non-financial support from Biocodex, BGP Products, Chiesi, Hipp, Humana, Mead Johnson Nutrition, Merck Sharp & Dohme, Nestle, Norsa Pharma, Nutricia, Roche, Sequoia Pharmaceuticals, and Vitis Pharma, outside the submitted work, and also grants, personal fees and non-financial support from Nutricia Research Foundation Poland, also outside the submitted work. The remaining authors declare that the research was conducted in the absence of any commercial or financial relationships that could be construed as a potential conflict of interest.

## Publisher's Note

All claims expressed in this article are solely those of the authors and do not necessarily represent those of their affiliated organizations, or those of the publisher, the editors and the reviewers. Any product that may be evaluated in this article, or claim that may be made by its manufacturer, is not guaranteed or endorsed by the publisher.
